# The development of a roadmap for Health Technology Assessment implementation in Moldova

**DOI:** 10.12688/f1000research.146025.1

**Published:** 2024-05-20

**Authors:** Gavin Surgey, Ghenadie Curocichin, Maria Garabajiu, Tanja Novakovic, Adrian Pana, Wija Oortwijn

**Affiliations:** 1 Global Health Priorities, RadboudUMC, Nijmegen, 6525 GA, The Netherlands; 2 Health Economics and HIV and AIDS Research Division, University of KwaZulu-Natal, Durban, South Africa; 3 Nicolae Testemițanu State University of Medicine and Pharmacy, Chișinău, 2004, Moldova; 4 ZEM Solutions Ltd, Belgrade, Serbia; 5 Independent, Bucharest, Romania

**Keywords:** HTA, Health Technology Assessment, UHC, Priority-setting; Roadmap; Moldova; EDPs; Evidence-informed deliberative processes

## Abstract

**Background:**

Moldova strives for universal health coverage (UHC) and considers health technology assessment (HTA) an important policy instrument to inform the choice of services that should be covered to progressively realize UHC. It plays a key role in determining which technologies are coved, considering various dimensions often including cost-effectiveness, budget impact, and feasibility. This paper reports on work undertaken to develop a roadmap for HTA implementation, using evidence-informed deliberative processes (EDPs) as the guiding framework.

**Methods:**

Between 2020 and 2022, we undertook several activities that informed the roadmap. We conducted a needs assessment and an assessment of European HTA best practices using a combination of desk research, interviews and surveys. We then conducted a document review of six selected HTA systems, complimented by expert interviews from three neighbouring countries.

**Results:**

The roadmap provided a suggested HTA process, which included instructions on how to execute the different steps of the EDP framework to enhance the legitimacy of HTA-informed decision-making. The roadmap encompasses several aspects such as how to organize stakeholder involvement, how to identify and operationalize decision criteria, and how to make the decision process transparent. Guidance was given to the country on establishing a legal framework for HTA; a communication strategy; strengthening capacity and leveraging HTA expertise. The country was also given tailored advice on the positioning of the HTA-agency, first as an entity within the Ministry of Health or the National Agency of Public Health but with the long-term vision for it to be independent of any state institution.

**Conclusions:**

Despite several challenges, including a partial disruption because of the Covid-19 pandemic, the development of the roadmap for HTA implementation was completed and agreed upon by the Ministry of Health in Moldova. This work has helped motivate and support the country in HTA institutionalization.

## Introduction

Like many countries worldwide that aim to achieve universal health coverage, Moldova views health technology assessment (HTA) as a significant policy tool as it plays a key role in determining which technologies are coved, considering various dimensions often including cost-effectiveness, budget impact, and feasibility.

HTA is widely used internationally to inform resource allocation decisions. It is defined as “a multidisciplinary process that uses explicit methods to determine the value of a health technology at different points in its lifecycle. The purpose is to inform decision-making to promote an equitable, efficient, and high-quality health system.” ^1^

While HTA is still in its infancy in Moldova, the country has been supported by partners for the past years to improve their capacity for HTA. This paper reports on the process used to develop a roadmap for HTA implementation and activities with the overall objective to establish the most suitable HTA system in Moldova. The activities executed between October 2020 and June 2022 were funded by the World Bank and supported by the Ministry of Health (MOH) and the National Health Insurance Company (CNAM) in Moldova.

We employ evidence-informed deliberative processes (EDPs) as the guiding framework ^2^ as well as experience with frameworks tested in other countries. The practical guidance on EDPs was developed by Radboudumc ^3^ and provides recommendations of how to implement each step of the decision-making process of benefits package design. The guide takes the current decision-making context in a country as the starting point, offering practical support depending on the country’s level of HTA development.

### Country context

Moldova is one of the lowest income countries in Europe, with a Gross National Income (GNI) per capita of $4,570 in 2020. The country heavily relies on the agricultural sector, and its GDP per capita is estimated to be $5,231 in current US dollars. ^4^ The constrained economic situation in Moldova can lead health system challenges such as funding shortages, limitations in service provision and increased out–of–pocket payments for users.

In 2019, the portion of GDP allocated to healthcare spending was 3.8%, which fell below the average of 6% for the European Union (EU) and 5% for South-Eastern Europe (SEE). However, it exceeded the average of 2.7% for lower-middle-income countries (LMIC) within the WHO European Region. ^5^ Public spending on health as a share of GDP increased to 4.8% in 2020 likely due to an increase in spending related to Covid-19. The annual GDP change for the Republic of Moldova was –7% between 2019 and 2020 (World Bank, 2022). ^6^

A significant number of people in the Republic of Moldova still lack access to affordable and high-quality essential healthcare services. To address this issue, the Moldovan government established a national purchasing agency (CNAM), which aimed to pool individual and state contributions. The objective was to create a strong foundation for gradually expanding the coverage of healthcare services and medications provided by CNAM to a larger portion of the population. ^7^

While this initiative has resulted in improved accessibility to healthcare services and a decrease in unmet needs, Moldova still experiences a higher incidence of catastrophic health expenditure compared to other countries in the WHO European Region. This is primarily due to persistent gaps in healthcare coverage. ^8^ Although CNAM has been progressively increasing the number of medicines it covers, there are still essential medications that are not included, and outpatient medicine coverage remains limited.

The entitlement to publicly funded benefits under mandatory health insurance in Moldova is defined by the 1998 Law on mandatory health insurance (No.1585-XIII). The purpose of mandatory health insurance is to guarantee equal access to healthcare for all residents, who are obliged to be covered by CNAM.

## Methods

The framework that we used for developing an HTA roadmap concerned evidence-informed deliberative processes (EDPs), combined with frameworks tested in other countries. ^9^ EDPs ^3^ can be used to guide countries to improve their decision-making processes, ^10^^,^^11^ and have been implemented successfully in a range of low, middle and high-income countries. ^12^^,^^13^ The core concept of EDPs centers around legitimacy and is broken down into four key elements, with stakeholder involvement and transparency being of utmost importance. The EDP framework offers a pragmatic and systematic approach tool for priority setting in health. Its primary objective is to enhance the credibility of benefit package choices and associated results, such as population health and financial risk mitigation. The activities encompassed in this project includes a needs assessment, an assessment of best practices and the development of the roadmap.

### Needs assessment

We conducted a needs assessment to understand the decision-making context in which HTA could be used, stakeholders and their (potential) roles and, the capacity and skills for HTA within the country. The needs assessment consisted of three building blocks namely: a situational analysis, stakeholder analysis and a capacity and skills assessment through which we conducted interviews and surveys. To conclude the phase of the needs assessment, a virtual workshop was held with participants to show preliminary results from the needs analysis and to use the opportunity to conduct some capacity strengthening around HTA. Data collection instruments were developed and approved both by the Moldova National Ethical Committee (Date 22.12.2020 Nr. 1023) and Nicolae Testemitsanu SUMPh Committee for Research Ethics (Date 28.12.2020 Nr. 2). Data was collected between April– May 2021.

A list of potential participants was compiled through an online search and our knowledge of the situation in the country. The online search consisted of reviewing the MOH website, National Health Insurance (NHIC) website and a general Google search. We classified all stakeholders according to the 7Ps (policymakers, payers, product makers, principal investigators, patients and the public, providers, and purchasers) to ensure that we had a representative sample of each stakeholder. ^14^ Subsequently, our list of potential respondents was reviewed and revised by the World Bank and the MOH and an official letter of request to participate in the study was provided.

### Situational analysis

The situational analysis aimed to establish the feasibility of EDP application in Moldova. We conducted a desktop review using standard health indicators and the list of services currently included in the MHI to match supply and demand. This was to help understand data availability relevant to HTA development, priority setting, and evidence-informed decision making; to help understand the current policy landscape that could support the institutionalisation of HTA, and to describe relevant stakeholders and the political landscape in Moldova.

In parallel, we collected views through semi-structured interviews with identified stakeholders to understand their knowledge of, position regarding, and interest in HTA.

The interview protocol ^15^ was based on Oortwijn et al. ^16^ which consisted of elements that reflect each step of the EDP framework and the contextual factors for HTA development.

### Stakeholder analysis

The stakeholder analysis aimed to identify and analyse potential stakeholder roles. We adapted and applied a stakeholder mapping tool ^15^ developed by Vlad. ^17^

The different perspectives and roles of various stakeholders would help understand who might influence and shape governance structures and legislative measures to support HTA institutionalisation and how far evidence-building tools such as HTA would be taken into account in decision-making.

### Capacity and skills assessment

Through a survey, ^15^ we assessed the available capacity and skills for HTA in Moldova of both ‘do-ers’ and users of HTA, although many of the respondents completed the survey in the presence of researchers. The survey was based on an existing set of tools tested out in other country assessments, ^18^ complemented with literature. ^19^

### Assessment of best practices

To draw lessons in implementing HTA in Moldova we assessed best practices in HTA which involved a mapping of HTA systems in Europe. For this purpose, we undertook literature reviews and conducted interviews with key stakeholders.

### Review of HTA systems

We first conducted a review of ‘comparative analyses’ from published literature in the last 5 years and then a focused analysis of six countries’ HTA systems.

For the focused analysis we selected three early adopters (or mature) and three ‘recent’ (or nascent) adopter countries based on our review and experience. These were England, Scotland and Germany (early adopter countries) and Romania, Poland and Ukraine (recent adopters). ^20^

The focused analysis examined each country’s HTA system according to each step of the EDP framework: A. installing an advisory committee; B. selecting decision criteria; C. selection of health technologies for HTA; D1. scoping; D2. assessment; D3. appraisal; E. communication and appeal; and F. monitoring and evaluation. ^3^ For this, we reviewed key documents and websites of HTA bodies to assess practices both in terms of HTA institutional design and the HTA process.

### Interviews with selected country key experts

For a selection of countries that could serve as an inspiration for Moldova, we conducted in-depth, semi-structured interviews with key experts and policymakers who were knowledgeable about their countries’ HTA system and to consider the transferability of the lessons learned to Moldova. We identified 3-5 stakeholders per country, via our networks, and invited them for a telephone interview.

The interview focused on the establishment of HTA in the respective country, and the steps of EDPs where we validated and/or updated the findings from the literature review of HTA systems.

### Development of a roadmap

Semi-structured in-depth interviews were conducted with senior Moldova representatives including three from the Ministry of Health, and four from the National Agency for Public Health to learn more about the Moldavian health system and the vision that for HTA in the country.

A workshop with stakeholders was held to validate the lessons to be drawn from the review and the neighbouring countries. The workshop also aimed to discuss key elements from other countries that would work in Moldova and where more work would be needed, both in terms of HTA institutionalisation and HTA processes.

The workshop also aimed to validate the recommendations developed and help create buy-in and legitimacy for the implementation plan to be developed.

Using a synthesis of findings from the needs assessment, assessment of best practices, and further discussions with key stakeholders from Moldova, a roadmap for HTA in Moldova was developed.

## Results

### Overview of respondents

There were 61 participants initially identified, and 55 were agreed to be contacted by e-mail. Potential participants who did not respond to the e-mail were sent a reminder 2 weeks after the initial invitation. On the second follow-up, six participants explicitly abstained from participation – due to several reasons including changing positions and a feeling of not being entitled to make decisions as their positions were solely technical in nature. A further six potential participants did not reply to the email nor phone calls. Respondents who accepted the invitation by e-mail were contacted by phone for scheduling the interview or agreed to complete the survey independently. An additional 3 technology producers were contacted through our networks as we had difficulty securing participants from this stakeholder category. Of these, 2 were successfully interviewed.

The number of respondents for each (7P) stakeholder category who participated are shown in Table 1.

**Table 1.** Overview of respondents.

Stakeholder category	Activity participated in		
	Situational analysis	Stakeholder analysis	Capacity and skills assessment
Policymakers	7	6	4
Payers	5	5	5
Product makers	2	2	2
Principal investigators	5	5	7
Patients and the public	2	2	2
Providers	3	3	4
Total respondents	24	23	24

Concerning the interviews with selected country key experts, we aimed to get 3-5 respondents (in Poland, Romania and Ukraine). However, it was challenging to get a timely response from more than 5 so we decided to interview at least 3 experts in each country, representing different stakeholder groups ( Table 4). We did not reach out to additional stakeholders once we had a minimum of 3 stakeholders per country.

### Situational analysis

Very few respondents felt that they were very familiar (33%) with HTA, although some (58%) indicated they had partial familiarity. When questioning respondents regarding the potential involvement of their organization in HTA, 17 respondents (71%) indicated that they or their organizations would have involvement in HTA indicating that this was the correct target audience for the survey.

Respondents indicated that Moldova is in the nascent phase of applying (elements of) EDPs for legitimate health benefits package design. There were, however, several elements that were shown to already be in place in Moldova which is in line with the HTA process including decision-making (advisory) committees with a level of legitimacy through government (legal) orders. The advisory committee for medicines called the “Council for Compensated Medicines” makes decisions on which medicines to include in their essential medicines list.

For all the steps and elements stipulated in the EDP framework, we probed whether there was a need for further guidance. Unsurprisingly, the majority of respondents felt that guidance was needed for all steps.

### Stakeholder analysis

We received complete responses from a total of 23 individuals who were either interviewed or completed a survey form before the interview which we followed up on afterwards.

The stakeholder analysis helped identify relevant stakeholders and their role or level of importance in priority setting in Moldova. This is shown in Table 2.

**Table 2.** Stakeholders and importance in priority setting in Moldova.

Stakeholder category	Key role	Important	Not important
Ministry of Health	87%	9%	0%
Ministry of Finance	39%	35%	13%
Any other central government body	20%	60%	10%
Elected decision-making committee	30%	61%	0%
Health insurers (government/private)	52%	35%	4%
Academic or research institutions	9%	70%	0%
External donors	13%	65%	13%
NGOs	0%	77%	5%
Healthcare providers (e.g. public/private hospitals)	18%	59%	9%
Clinicians	9%	70%	9%
Professional organisations (e.g. medical associations)	9%	78%	0%
Patient and/or carer organisations	4%	70%	13%
Pharmaceutical and/or devices industry	9%	74%	4%
Civil Society	0%	70%	13%
Any other group not mentioned	0%	22%	9%

We found that respondents reported several stakeholders involved in priority setting and that there is seemingly a collaborative environment between stakeholders.

In trying to understand the *role of politicians/appointed decision-makers in defining the benefits package,* respondents stated that their main role is around decision-making and approval of decisions. They noted that they have the responsibility for applying changes to the benefits packages such as updating with new medicines. There were no stated political tensions or disagreements. Civil servants and non-health professional decision-makers had similar roles, but there their specific role in the process was not clear. When it came to an understanding of the process of defining the benefits package it was reported that the MOH was responsible for coordinating the procedure with CNAM (the health insurer) and that there were appointed committees responsible for this, but the overall procedure was not clear to respondents.

The Center for Centralized Public Procurement in Healthcare (CAPCS) was stated as having responsibility for the procurement of medical products. The Agency for Medicines and Medical Devices (AMDM) was noted as having responsibility for negotiating prices, although there was no clear consensus among respondents. Respondents mentioned several stakeholders as having responsibility for pricing.

When it comes to decision-making, respondents felt that *Patients or carer groups and the public* were given a voice either partially (33%) or fully (27%). However, voting on decisions is restricted to only a few key policy-makers and this means that there is mostly consultation of stakeholders and very little participative decision-making.

### Capacity and skills assessment

The majority of respondents to the survey had expertise in the field of public health and management. There was an equal split between those who classified themselves as decision-makers (users) or researchers (producers/do-ers).

We found that half of the respondents (50%) were part of, or had previously belonged to a government advisory committee of some sort and thus showed an average or above-average level of competence in related leadership or management skills. There was a good percentage of respondents (81%) who had experience with, and specific skills in conducting academic research and writing publications. This could partly be explained by the fact that there was a significant proportion of respondents (21%) who were employed by a university/research organisation.

Respondents rated their technical skills related to HTA as being moderate with more than half showing that they had specific experience in the majority of categories. Some respondents (31%) stated that they had read HTA reports from other countries, although there was a lower amount (15%) who stated that they had used the results from other countries.

However, when it came to specific technical skills (such as economic evaluations) the majority indicated a low level of confidence. Few showed confidence in conducting appraisals which is a key element of the HTA process where the results of the assessment need to be interpreted from a broader perspective and where recommendations are developed to inform decision-making.

Results relating to the capacity and skills assessment can be found in Table 3.

**Table 3.** Familiarity, experience and knowledge related to HTA.

Familiarity of HTA					
	Not at all	Low	Moderate	Fairly familiar	Very familiar
How familiar are you with HTA?	4.00%	20.00%	60.00%	8.00%	8.00%

Level of experience in HTA				n	%
Produced or used/referred to an hta report		No, never produced or used an HTA report		8	30.80%
		Yes, I only read the report from another country		8	30.80%
		Yes, I read and used the results from the report from another country		4	15.40%
		Yes, I have produced/contributed to an HTA report		6	23.10%
Ever used, or referred to a systematic review or other types of evidence synthesis (e.g. rapid review, meta-analysis)?		Yes		20	76.90%
		No		6	23.10%
Ever undertaken a (systematic) review or a meta-analysis of the clinical/medical literature		No		9	34.60%
		Yes, 1-5 reviews		9	34.60%
		Yes, 6-10 reviews		3	11.50%
		Yes, more than 10 reviews		5	19.20%
Ever undertaken an economic evaluation?		Yes		14	53.80%
		No		12	46.20%
How many economic evaluations?		1-3		8	4-5%
		4-5		2	14.30%
		6-7		1	7.10%
		≥ 8		3	21.40%
Type of analysis		Cost-benefit analysis (CBA)		8	57.10%
		Cost-effectiveness analysis (CEA)		10	71.40%
		Cost-utility analysis (CUA)		3	21.40%
		Other (please specify)		0	0.00%
Ever used the results of an economic evaluation?		Yes		10	38.50%
		No		16	61.50%
Ever undertaken health-related quality-of-life studies? If yes, how many?		No		12	46.20%
		1-3		13	50.00%
		4-5		1	3.80%
Ever used the results of health-related quality-of-life studies?		Yes		17	65.40%
		No		9	34.60%
Ever undertaken studies to assess ethical, social, cultural and legal issues; organizational and environmental aspects and/or implications for the patients, relatives, caregivers and the population		None, 0		11	42.30%
		1-3		13	50.00%
		4-5		2	7.70%
Ever used the results of studies that assess ethical, social, cultural and legal issues; organizational and environmental aspects and/or implications for the patients, relatives, caregivers and the population		Yes		18	69.20%
		No		8	30.80%

Level of knowledge and confidence in conducting/using HTA activities					
	No knowledge and confidence	Heard of it but not confident in doing any of it	Slightly confident: Have some understanding in this area	Confident: Can interpret results already produced in this area	Expert: Can produce research and analysis in this area
Determining if the PICO (TS) (population, intervention comparator, outcome, (type of study, timing and setting) presented is correct	23.10%	15.40%	34.60%	19%	8%
Systematic literature reviews	3.90%	23.10%	26.90%	27%	19%
Differentiation between cost minimisation, cost-effectiveness, cost-utility and cost-benefit	0.00%	30.80%	30.80%	31%	8%
Determination of the impact of decisions on health inequity	11.50%	34.60%	19.20%	31%	4%

**Table 4.** Stakeholders interviewed for assessment of best practices.

	Poland	Romania	Ukraine
Policymakers	2	2	2
Payers	1	-	-
Principal investigators	1	1	2
Total	4	3	4

### Workshop

Twenty-five individuals attended the workshop primarily from the MOH and CNAM. Preliminary results from the needs assessment were shown and discussed. The role of stakeholders was highlighted and there was discussion about how to further engage other stakeholders to actively participate in the process. Participants agreed that the level of understanding around HTA was too weak which might explain the current lower level of engagement around HTA.

Capacity strengthening was also undertaken to further sensitize on UHC, and HTA concepts and methods. EDPs were presented as a practical approach to implementing HTA in Moldova, and how these processes could be used to promote legitimate decision-making on the path to UHC.

### Review of HTA systems

A review of ‘comparative analyses’ from published literature in the last 5 years shows that the majority (29 out of 31 countries, 94%) of countries (where information was available) use some elements of HTA to support decision-making about the use of pharmaceuticals. HTA activity was found to be lower for non-pharmaceutical health technologies with 71% of countries (22 out of 31 countries) using HTA to support decision-making.

The comparison of the six selected countries, early adopters (England, Scotland and Germany) and recent adopters (Poland, Romania and Ukraine) show that:

• In early adopter countries, stakeholders are involved in nearly all HTA processes which is not the case in recent adopter countries. It appears that there is no stakeholder engagement in most steps;
• Early adopters (Germany and England) conduct explicit monitoring and evaluation of decisions, while recent adopters seem not to monitor or evaluate the results of HTA decisions;
• Training of stakeholders exists in early adopter countries and Poland, but not in Ukraine and Romania;
• Assessment and appraisal phases are the most developed processes across all countries, with methodological HTA guidelines in place in England, Poland and Ukraine. The Polish appraisal process seems to have the highest level of transparency compared to the other two recent adopters (Romania and Ukraine).

### Interviews with selected country key experts

Discussions with key experts gave insight as to some of the reasons the country had introduced HTA and some conducive factors. How HTA was set up and other recommendations for newly establishing HTA in a country.

The main reasons respondents gave for introducing HTA were economic concerns such as budget around spending on health and the need for cost-containment. Some conducive factors to introducing HTA was political will supported by new legislation to enforce HTA. Other key success factors that respondents noted included building support for various stages in the establishment of HTA and having a close collaboration with all stakeholders including industry and patients throughout the process.

Experts also gave some recommendations to Moldovan policymakers on establishing HTA. These included ensuring the involvement of multiple stakeholders and building support for the various stages of HTA and establishing a solid methodology and processes containing all the steps for HTA. Experts also advised on the importance of creating an HTA assessment body independent of state authorities to make decisions regarding reimbursement and building the right legislation around this. It was also recommended to continuously invest and build the institutional capacity of those involved in HTA in the country.

### Development of a roadmap

A final workshop was held with approximately 25 individuals primarily from the MOH and CNAM. Experiences and best practices were shared with presenters who lead HTA activities in Poland and Ukraine. Presentations and discussions were held on the current situation of Moldova (issues and challenges) and how the country can move towards the establishment of an HTA system.

During the workshop it was discussed where the HTA unit should be based - it was agreed that for now it should start with either the MOH or the National Agency of Public Health but the long-term vision would be for it to be independent of any state institutions. The main purpose agreed upon during the workshop was for the HTA unit to focus on developing the benefits package and keeping the Essential Medicines List up to date.

The development of the roadmap took into consideration all the findings and other discussions that were held with Moldavian stakeholders instrumental in the design of HTA. The roadmap covered the HTA institutional design in terms of the responsibilities of participating organizations in implementing HTA evidence into policy. The roadmap also gave details of a proposed HTA process including guidance on how to implement the steps of the EDP framework. This included providing suggestions on how to effectively organize stakeholder involvement, how to potentially identify and operationalize decision criteria, and how to best make the decision process transparent. Specific guidance was given to the country on establishing a legal framework for HTA; a communication strategy; strengthening capacity and leveraging HTA expertise. Recommendations given were in line with the 2020 WHO report “Principles of health benefit packages”, ^21^ and the “roadmap for systematic priority setting and HTA” by Castro, Kumar, Suharlim et al. ^22^

## Discussion

This paper outlines the components that can support the successful development of a roadmap for HTA implementation in Moldova. HTA leads to better informed benefit package and improved access to essential services which is critical for the achievement of UHC. ^23^^,^^24^ Through this research, we find that there is a high demand and appetite by all stakeholders for HTA in the country. It showed that there is a strong political will, but this is driven by very few within the government making the success of Moldova very dependent on a few individuals who have already stretched agendas. There are a few stakeholders that have received some training on HTA however, there is still a lot more sensitization needed across all stakeholder groups. There are limited resources including human and financial resources but also a limited amount of country-specific data. However, the country has some decision-making systems and processes that can be harnessed for HTA. We believe that any additional guidance on existing processes will only strengthen the processes for Moldova.

Currently, there are a handful of activities taking place to establish HTA in the country which were known by respondents including demonstration projects and some training courses in the field of HTA. However, given the number of stakeholders (individuals and departments) who were reported to have kickstarted the establishment of HTA, we might expect to see more activities currently taking place.

The surveys conducted did have some shortfalls, for example when measuring skills, we did not provide a definition of terms meaning respondents could have interpreted these differently. Results are therefore prone to measurement bias. We saw this when asking knowledge questions, i.e. asking a respondent to rate their knowledge on a topic and then asking them a verification question on that topic. We found that they did not always rate their knowledge correctly. As such, there is a need for creating awareness, training and education in HTA. Stronger technical skills are needed in using different types of evidence (reviewing evidence) and interpreting economic evaluations.

Involving multiple stakeholders is expected to enhance the credibility of Moldova's decision-making process. ^3^ Two successful workshops were conducted with stakeholders to ensure alignment of objectives and consensus on advancing Health Technology Assessment (HTA) in the country. In these sessions, stakeholders reached a mutual understanding regarding the rationale, goals, and scope of HTA initiatives. Institutionalizing HTA and enhancing the legitimacy of this process will reinforce the overall credibility of decision-making and foster greater transparency, given the comprehensive involvement and informed status of numerous stakeholders.

The overall project and development of the roadmap did face a challenge in that almost all of the work was developed remotely given the disruption due to COVID-19. Implementation of the roadmap will likely also experience delays due to pressing issues related to COVID-19 and the current situation in Ukraine. Moldova has, however, started to take some of these recommendations forward in developing capacity-strengthening options with local universities. Building skills in this area will help drive the process of institutionalization of HTA in Moldova.

An important contribution of this paper is to demonstrate how countries can institutionalise HTA for the achievement of UHC. Critical to the success is the continuous political will and determination of the government ^25^ of coupled with comprehensive stakeholder engagement. ^26^^,^^27^ From here, HTA should be linked to policy decision-making supported by the HTA legislation with a permanently financed core team focused on HTA. ^28^ At the same time, developing formal HTA training capacity so that over time, skills and expertise are built. This needs to be accompanied by continuous awareness raising among users of HTA. International collaboration with other countries that are further in the HTA process as well as international networks such as HTAi and INAHTA can support Moldova in the implementation of the roadmap.

### Ethical considerations and consent

Ethical approval for this study was obtained from the National Committee for Ethical Expertise of Clinical Trial (NCEECT/1023/22.12.2020) on 22.12.2020 and Nicolae Testemitanu State University of Medicine on and Pharmacy (94) on 21.12.2020. All participants gave either verbal or written informed consent depending on the nature of the survey. Verbal informed consent was used when participants were supported in completing a survey form, or when an survey form was completed on their behalf.

## Data Availability

OSF: Roadmap for HTA implementation in Moldova [dataset] OSF: DOI 10.17605/OSF.IO/NPKBF File ‘Moldova HTA Roadmap data’ contains individual underlying data for each 3 surveys File ‘1. Instrument - Situational Analysis.docx’ contains situational analysis instrument File ‘2. Instrument - Stakeholder Analysis.docx’ contains stakeholder analysis instrument File ‘3. Instrument - Capacity Skills Assessment.docx’ contains capacity skills assessment instrument File ‘4. Review of early and recent adopter countries against EDP steps.docx’ contains individual country level results All data underlying the results are available as part of the article and no additional source data are required. Data are available under the terms of the CC0 licence *CC0 1.0 Universal*
