# Supplementary material for: The development of a roadmap for Health Technology Assessment implementation in Moldova
Source: F1000Res. 2024 Oct 21;13:518. Originally published 2024 May 20. [Version 2] doi: 10.12688/f1000research.146025.2 (PMC11502998; doi:10.12688/f1000research.146025.2)
Supplement: Peer review history

# Reviewer report: Reviewer response for version 2

**DOI:** 10.5256/f1000research.173342.r334159

**Published:** 2024-11-25

**Authors:** Eleanor Grieve

**Affiliations:** 1 University of Glasgow, Glasgow, Scotland, UK

Thank you for the opportunity to review this research into the process of initiating HTA in a country new to these systems.  A very thorough needs assessment was undertaken (situational analysis, stakeholder analysis and capacity / skills assessment); an assessment of European HTA best practices using a combination of desk research, interviews and surveys;  a document review of six selected HTA systems; and expert interviews from three neighbouring countries.  The country was given advice on the positioning of the HTA-agency.

 I don’t have too much to add to the other two thoughtful reviews.  My main thoughts are around the benefit of taking a deeper dive into each type of stakeholder, or perhaps specifically industry and academia/research (they are both listed in Table 2). 

 The emphasis is mostly on government clearly as policy/decision makers but for a country embarking on an HTA journey, it would be good to understand HTA from the perspective of the country’s relationships with pharma/industry and research. There is some albeit brief mention of both. 

 For pharma, the Center for Centralized Public Procurement in Healthcare is stated as having responsibility for the procurement of medical products, and the Agency for Medicines and Medical Devices for price negotiations.  More information here would be good, for example, are price negotiations based purely on budgetary concerns or value / cost-effectiveness too?  What is the process?  Where would HTA sit in this process?

 Likewise, with research, it is briefly state that there is a limited amount of country-specific data.  Is this referring to routinely collected health monitoring data or to research generating country-specific evidence.

 HTA needs to be linked into both research/evidence generation and industry as well as government and so perhaps more on the former two could be added or explored if out of scope in this study.

Is the work clearly and accurately presented and does it cite the current literature?

Yes

If applicable, is the statistical analysis and its interpretation appropriate?

Not applicable

Are all the source data underlying the results available to ensure full reproducibility?

No source data required

Is the study design appropriate and is the work technically sound?

Yes

Are the conclusions drawn adequately supported by the results?

Yes

Are sufficient details of methods and analysis provided to allow replication by others?

Yes

Reviewer Expertise:

Health Economics and Health Technology Assesment

I confirm that I have read this submission and believe that I have an appropriate level of expertise to confirm that it is of an acceptable scientific standard.

# Reviewer report: Reviewer response for version 2

**DOI:** 10.5256/f1000research.173342.r333444

**Published:** 2024-10-24

**Authors:** Christian Suharlim

**Affiliations:** 1 Management Sciences for Health Medford (Ringgold ID: 482848), Medford, Massachusetts, USA

No further comments. Thank you for the clarification

Is the work clearly and accurately presented and does it cite the current literature?

Yes

If applicable, is the statistical analysis and its interpretation appropriate?

Partly

Are all the source data underlying the results available to ensure full reproducibility?

No

Is the study design appropriate and is the work technically sound?

Yes

Are the conclusions drawn adequately supported by the results?

Yes

Are sufficient details of methods and analysis provided to allow replication by others?

Partly

Reviewer Expertise:

Health Technology Assessment in LMICs; economic evaluation

I confirm that I have read this submission and believe that I have an appropriate level of expertise to confirm that it is of an acceptable scientific standard.

# Reviewer report: Reviewer response for version 1

**DOI:** 10.5256/f1000research.160056.r288867

**Published:** 2024-06-25

**Authors:** Kanchan Mukherjee

**Affiliations:** 1 Centre for Health Policy, Planning and Management in the School of Health Systems Studies, Tata Institute of Social Sciences, Mumbai, India

This paper documents the process used for developing a road map for HTA implementation in Moldova. The methodology involved using the EDP framework, which has been applied before, and thus provides transparency with scope for replicability. The steps included needs assessment (situational analysis, stakeholder analysis, capacity assessment), which provided an internal contextual assessment of the HTA situation in Moldova. This was complemented by assessment of best practices, and neighboring countries key expert interviews, which provided the external contextual analysis. Overall, the above activities suggest a holistic approach to the situation, while acknowledging the contextual influence. Contextualisation of HTA is extremely important and has also been reported in literature in creating a road map for HTA in India, which is a low-middle-income country (like Moldova) (Mukherjee K et al, 2017) [Ref 1]

 The methodology followed is comprehensive and justifiable given the objective of this exercise. It also reflects a well thought out process which is systematic and specific, while acknowledging the limitations of such a process. The conclusions and discussion reflect the positive aspects (high demand by stakeholder) as well as the challenges (few policy champions for HTA, low HTA capacity).

 India also encountered a similar situation like Moldova, when the process of HTA was first conceived at the political level and continues to face similar challenges in its implementation. From my HTA experience in the  Indian context, I am sharing a few learnings, which maybe useful for Moldova:

1. Political institutionalization is extremely important, but the HTA body should be an autonomous entity to avoid political interference in the HTA process.
2. Academic institutionalization of HTA through local universities is a sustainable model for capacity building in HTA. Hence, it may also be useful to consider academic institutionalization of HTA in local universities in the form of Masters/PhD programme or short term training programmes, in which policy makers/implementers, can also be nominated to be trained in the HTA process. This model has been implemented in India since 2010 and has resulted in a demand creation in this niche area as well as increased sensitization and awareness among policy makers about HTA (Mukherjee K, et al 2020) [Ref 2].
3. It may be useful to have small pilot HTA projects (‘mini-HTA’) at the initial stages to provide credibility, legitimacy and acceptance by various stakeholders. Hence, simple incremental steps in HTA based on existing resources and decision making mechanisms maybe useful before using HTA for complex issues.
4. The HTA ecosystem should be an open system receptive to learnings (internal and external), adaptive and responsive to evolving challenges over time. Hence, it would be useful to have a follow up review to assess the extent of implementation of HTA in Moldova.

To conclude, I congratulate the team for their efforts in this exercise, which by itself would have resulted in sensitization of stakeholders and started the first step towards HTA implementation in Moldova.

Is the work clearly and accurately presented and does it cite the current literature?

Yes

If applicable, is the statistical analysis and its interpretation appropriate?

Not applicable

Are all the source data underlying the results available to ensure full reproducibility?

Yes

Is the study design appropriate and is the work technically sound?

Yes

Are the conclusions drawn adequately supported by the results?

Yes

Are sufficient details of methods and analysis provided to allow replication by others?

Yes

Reviewer Expertise:

HTA, Health Policy, Public Health

I confirm that I have read this submission and believe that I have an appropriate level of expertise to confirm that it is of an acceptable scientific standard.

## References

[1] : Health technology assessment: A potential roadmap for India. *International Journal of Medical Science and Public Health* .2017; 10.5455/ijmsph.2017.1269225012017 10.5455/ijmsph.2017.1269225012017

[2] : A SMART framework for HTA capability development: Lessons from India. *Health Policy and Technology* .2020;9(1) : 10.1016/j.hlpt.2019.12.001 42-44 10.1016/j.hlpt.2019.12.001

# Reviewer report: Reviewer response for version 1

**DOI:** 10.5256/f1000research.160056.r288862

**Published:** 2024-06-14

**Authors:** Christian Suharlim

**Affiliations:** 1 Management Sciences for Health Medford (Ringgold ID: 482848), Medford, Massachusetts, USA

Thank you for the opportunity to review this paper. The authors did an excellent job in describing, in detail, the activities conducted in order to assess the needs, propose improvements, and develop the HTA Roadmap. I suggest a few revisions below:

1. Authors described in [Results-Stakeholder analysis] "However, voting on decisions is restricted to only a few key policy-makers and this means that there is mostly consultation of stakeholders and very little participative decision-making." I would have liked to see a deeper dive into this aspect in the Discussion section, e.g., Does this align with what researchers would expect ex-ante for a country with Moldova's history and government? Does the comparative analysis shed insight on how Moldova compares in this regard to other post-Soviet states? How is this improved in the new roadmap?
2. Authors put quotation marks around the term comparative analyses. I wonder why this is the case. I would have liked to have more detail on how the 5-year published literature desk review was conducted under [methods] and a table of the findings under [results] and [Supplementary data]
3. In the introduction, the Authors specifically mention how Moldova is lower on the development level in Europe- as proxied by GNI/capita. However, the six focused countries are mostly high-income countries with vastly different development levels. Under [Discussion], I would like to see how authors synthesize the impact of the development level difference between these countries and Moldova and how it may impact the generalizability and applicability of a country with a much lower development level. In particular, it would be excellent if authors could comment on human resource capacity, e.g., human flight and brain drain, and how that may impact a country with such issues to meet the unfair expectations of the gold standards set by early adopters.
4. Under [Results-development of a roadmap] "Experiences and best practices were shared with presenters" - or "by presenters"?
5. Under [Discussion], the authors state, "It showed that there is a strong political will, but this is driven by very few within the government, making the success of Moldova very dependent on a few individuals who have already stretched agendas." I would have expected this to be the case in most countries with lower development levels.Does this align with the Authors' desk review / comparative analyses? If this is indeed the typical lay of the land in LMICs, is there a precedent to more positive wording (e.g., 'local champions'?)
6. The authors state their limitations in the survey. I would have liked to see if authors could synthesize their thinking on the current limitations with the desk review, interviews, sample size and selection, and interpretation of data.
7. The authors made a point about improving stakeholder involvement. However, I would have liked to see the intensity and extent of these 'new stakeholders' contributions. What (if any) decisions would have been different had they not been a part of the contribution? Beyond the cited theory and paper, in Moldova, how would their involvement improve the legitimacy of decisions? Were there explicit buy-ins and endorsements by the new stakeholders' affiliated organizations?
8. Lastly, this is a really well-done paper and process. The authors would benefit from more explicit statements that one of the strengths of this study is the multi-stage sequential mixed-method approach, which allowed them to assess needs methodically and offer solutions.

Congratulations for the excellent work and a job well-done!

 Best regards,

 Chris Suharlim

Is the work clearly and accurately presented and does it cite the current literature?

Yes

If applicable, is the statistical analysis and its interpretation appropriate?

Partly

Are all the source data underlying the results available to ensure full reproducibility?

No

Is the study design appropriate and is the work technically sound?

Yes

Are the conclusions drawn adequately supported by the results?

Yes

Are sufficient details of methods and analysis provided to allow replication by others?

Partly

Reviewer Expertise:

Health Technology Assessment in LMICs; economic evaluation

I confirm that I have read this submission and believe that I have an appropriate level of expertise to confirm that it is of an acceptable scientific standard, however I have significant reservations, as outlined above.

# Response

**Published:** 2024-10-10

**Authors:** Gavin Surgey

Thank you for the comments. We have responded to each of the comments in turn, and indicated where we have made additions to the paper. Thank you for the suggestions – we believe they have added to the overall paper.

*1. Authors described in [Results-Stakeholder analysis] "However, voting on decisions is restricted to only a few key policy-makers and this means that there is mostly consultation of stakeholders and very little participative decision-making." I would have liked to see a deeper dive into this aspect in the Discussion section, e.g., Does this align with what researchers would expect ex-ante for a country with Moldova's history and government? Does the comparative analysis shed insight on how Moldova compares in this regard to other post-Soviet states? How is this improved in the new roadmap?*

 Ans: We have added additional information related to:

- Alignment with expectations for Moldova – a paragraph was added in the discussion section
- Comparison to other post-Soviet states
- Improvements in the new roadmap and how learning from both regional peers and more established HTA systems, the new roadmap aims to accelerate Moldova's progress in this area.

2. *Authors put quotation marks around the term comparative analyses. I wonder why this is the case. I would have liked to have more detail on how the 5-year published literature desk review was conducted under [methods] and a table of the findings under [results] and [Supplementary data]*

 Ans: We attempted to make this more clear by adding additional information in the paper including: *We did not use the information to compare countries, but rather to give an overview of the HTA landscape in Europe.*

 We did not include a table of findings for the comparative analysis as this mainly served as background information for selecting countries for the focused analysis – and this was not the aim of the analysis. The table of findings for the focused analysis are presented in supplementary data file:

 File ‘4. Review of early and recent adopter countries against EDP steps.docx’ contains individual country level results

 An additional supplementary file was added (#5) to give a listing of the of the resources we used to gather the findings.

*3. In the introduction, the Authors specifically mention how Moldova is lower on the development level in Europe- as proxied by GNI/capita. However, the six focused countries are mostly high-income countries with vastly different development levels. Under [Discussion], I would like to see how authors synthesize the impact of the development level difference between these countries and Moldova and how it may impact the generalizability and applicability of a country with a much lower development level. In particular, it would be excellent if authors could comment on human resource capacity, e.g., human flight and brain drain, and how that may impact a country with such issues to meet the unfair expectations of the gold standards set by early adopters.*

 The countries were selected to serve as inspiration.

 Ans: We added information: We will select 2-3 countries that could serve as an inspiration for HTA development in Moldova. In choosing these countries, we **started to develop a selection tool** that includes the following criteria: similar health system financing and organization of health services delivery, although the perspective used in HTA may differ (health system verses societal perspective),  level of HTA development (intermediate/mature level, e.g. Croatia, Poland, Romania, Ukraine), level of EDP development or interested in EDP uptake (e.g. Kazakhstan, Ukraine).

*4. Under [Results-development of a roadmap] "Experiences and best practices were shared with presenters" - or "by presenters"?*

 Ans: Thanks for picking this up: “by presenters” is correct

*5. Under [Discussion], the authors state, "It showed that there is a strong political will, but this is driven by very few within the government, making the success of Moldova very dependent on a few individuals who have already stretched agendas." I would have expected this to be the case in most countries with lower development levels. Does this align with the Authors' desk review / comparative analyses? If this is indeed the typical lay of the land in LMICs, is there a precedent to more positive wording (e.g., 'local champions'?)*

 Ans: The terms "political will" and "local champions" are related but distinct. A "local champion" usually refers to an individual, not necessarily in government, who drives forward a specific agenda. In contrast, "political will" refers to the collective determination of a group of individuals within the government to pursue a particular course of action. While a local champion may lead or inspire efforts, political will reflects a broader, shared commitment within the governing body. We have not made any changes to this

*6. The authors state their limitations in the survey. I would have liked to see if authors could synthesize their thinking on the current limitations with the desk review, interviews, sample size and selection, and interpretation of data.*

 Ans: Some additional details relating to the limitations were added to the discussion section relating to the desk review. We also added additional paragraph on the multi-stage approach.

*7. The authors made a point about improving stakeholder involvement. However, I would have liked to see the intensity and extent of these 'new stakeholders' contributions. What (if any) decisions would have been different had they not been a part of the contribution? Beyond the cited theory and paper, in Moldova, how would their involvement improve the legitimacy of decisions? Were there explicit buy-ins and endorsements by the new stakeholders' affiliated organizations?*

 Ans: We appreciate the suggestion to further analyze the impact of expanded stakeholder involvement. You raise good questions about the tangible effects of including new stakeholders. We did not assess how specific decisions differ. We can at this time only refer to theoretical arguments and examples of other countries where stakeholder involvement improves legitimacy of decisions as well as buy-ins and endorsements. However, we agree this could be more thoroughly analysed as a separate piece of future work.

*8. Lastly, this is a really well-done paper and process. The authors would benefit from more explicit statements that one of the strengths of this study is the multi-stage sequential mixed-method approach, which allowed them to assess needs methodically and offer solutions.*

 Ans: Thanks for this suggestion, it has been added to the paper.

 We appreciate these insightful comments which will help strengthen both this paper and our ongoing work supporting HTA development in Moldova and other countries.

- **Are sufficient details of methods and analysis provided to allow replication by others?**

All the instruments and source data is available online. This is detailed in the “Data availability section”: OSF: Roadmap for HTA implementation in Moldova [dataset] OSF: DOI 10.17605/OSF.IO/NPKBF

  

- **If applicable, is the statistical analysis and its interpretation appropriate?**

Partly

 Descriptive analysis techniques were used. These methods are detailed in the paper with the individual results in the source data.

 OSF: Roadmap for HTA implementation in Moldova [dataset] OSF: DOI 10.17605/OSF.IO/NPKBF

  

- **Are all the source data underlying the results available to ensure full reproducibility?**

No

 All the instruments and source data is available online. This is detailed in the “Data availability section”: OSF: Roadmap for HTA implementation in Moldova [dataset] OSF: DOI 10.17605/OSF.IO/NPKBF

  

- **Are the conclusions drawn adequately supported by the results?**

Yes
